# Development of a wheat single gene FISH map for analyzing homoeologous relationship and chromosomal rearrangements within the Triticeae

**DOI:** 10.1007/s00122-013-2253-z

**Published:** 2014-01-10

**Authors:** Tatiana V. Danilova, Bernd Friebe, Bikram S. Gill

**Affiliations:** 1 Department of Plant Pathology, Wheat Genetics Resource Center, Kansas State University, Manhattan, KS 66506 USA; 2 Faculty of Science, Genomics and Biotechnology Section, Department of Biological Sciences, King Abdulaziz University, Jeddah, 21589 Saudi Arabia

## Abstract

*****Key message***:**

**A cytogenetic map of wheat was constructed using FISH with cDNA probes. FISH markers detected homoeology and chromosomal rearrangements of wild relatives, an important source of genes for wheat improvement.**

**Abstract:**

To transfer agronomically important genes from wild relatives to bread wheat (*Triticum aestivum* L., 2*n* = 6*x* = 42, AABBDD) by induced homoeologous recombination, it is important to know the chromosomal relationships of the species involved. Fluorescence in situ hybridization (FISH) can be used to study chromosome structure. The genomes of allohexaploid bread wheat and other species from the Triticeae tribe are colinear to some extent, i.e., composed of homoeoloci at similar positions along the chromosomes, and with genic regions being highly conserved. To develop cytogenetic markers specific for genic regions of wheat homoeologs, we selected more than 60 full-length wheat cDNAs using BLAST against mapped expressed sequence tags and used them as FISH probes. Most probes produced signals on all three homoeologous chromosomes at the expected positions. We developed a wheat physical map with several cDNA markers located on each of the 14 homoeologous chromosome arms. The FISH markers confirmed chromosome rearrangements within wheat genomes and were successfully used to study chromosome structure and homoeology in wild Triticeae species. FISH analysis detected 1U-6U chromosome translocation in the genome of *Aegilops umbellulata*, showed colinearity between chromosome A of *Ae. caudata* and group-1 wheat chromosomes, and between chromosome arm 7S#3L of *Thinopyrum intermedium* and the long arm of the group-7 wheat chromosomes.

**Electronic supplementary material:**

The online version of this article (doi:10.1007/s00122-013-2253-z) contains supplementary material, which is available to authorized users.

## Introduction

A wide variety of important genes were transferred to bread wheat, *Triticum aestivum* L., (2*n* = 6*x* = 42, genome AABBDD) from Triticeae species, but only few of them are exploited in wheat cultivars (reviewed by Friebe et al. 1996; Kilian et al. 2011). To select an optimal gene transfer strategy resulting in the introgression of only a small part of an alien chromosome with the gene of interest, but without undesirable traits and compensating for the replaced wheat chromatin, it is necessary to know the evolutionary distance, homoeologous relationships and degree of colinearity between wheat and alien chromosomes.

Several approaches were used to study the homoeology between chromosomes of hexaploid wheat and other Triticeae species. Gametophytic compensation test is based on non-viability of wheat male gametes with substituting alien non-compensating chromosomes. The analysis uses cytological or phenotypic screening of the progeny from crosses between wheat and set of wheat-alien double monosomic lines. The sporophytic compensation ability of alien chromosomes can be tested by measuring the fertility of wheat-alien substitution lines. Because of variation in the ability to substitute chromosomes from A, B and D genomes, this analysis requires the development of wheat-alien substitution lines for all three wheat genomes (Sears 1952; Dvorak 1980; Friebe et al. 1993).

Analysis of chromosome pairing in metaphase I of meiosis can also be used to determine the homoeology of wheat and alien chromosomes (Yang et al. 1996).

A comparative genetic analysis of alien chromosomes with wheat is another approach, which employs molecular or morphological markers. Only a few morphological, isozymic or seed storage protein markers are available for defining the homoeology of an alien chromosome, which limit the ability of the approach to reveal chromosomal rearrangements. (Yang et al. 1996; Zhang et al. 1998; Friebe et al. 1999; Qi et al. 2007; McArthur et al. 2012). Restriction fragment length polymorphisms (RFLPs) are the most informative wheat molecular markers (Qi et al. 2007). RFLPs were successfully used to develop high-resolution wheat genetic and physical maps (Qi et al. 2003, 2004), define homoeology of alien chromosomes and reveal their rearrangements relative to wheat (Devos et al. 1993a; Zhang et al. 1998; McArthur et al. 2012). Southern hybridization, however, is not a high-throughput technique and it employs radioisotopes (Qi et al. 2007). To perform genetic mapping of molecular markers on alien chromosomes it is necessary to develop and analyze segregating populations.

Fluorescence in situ hybridization (FISH) is a useful tool for physical mapping of chromosomes and for studying evolutionary chromosome rearrangements. FISH labeling of tandem repeats and microsatellites allows the identification of individual wheat chromosomes and chromosomes of related species (Mukai et al. 1993; Pedersen et al. 1996; Pedersen and Langridge 1997; Cuadrado and Schwarzacher 1998; Cuadrado et al. 2008a, b; Komuro et al. 2013). Genic sequences are highly conserved among Triticeae species (Feuillet and Keller 2002; Akhunov et al. 2003) and free of repeats, which makes them homoeolog-specific wheat FISH markers and potentially universal Triticeae markers. Single genes with size larger than 3 kb can be routinely localized on mitotic plant chromosomes by FISH, as was shown on maize, barley and wheat (Lamb et al. 2007; Danilova and Birchler 2008; Ma et al. 2010; Karafiátová et al. 2013). In our previous study, we used wheat cDNA of cytosolic acetyl-CoA carboxylase (*Acc*-*2*) and nine other full-length (FL) cDNAs together with tandem repeats as multicolor FISH probes to map *Acc*-*2* sequences, identify chromosomes and reveal chromosome rearrangements in wheat and its closest diploid and tetraploid relatives *T. urartu* Tumanian ex Gandilyan, *T. monococcum* L., *Ae. speltoides* Tausch., *Ae. tauschii* Coss., *T.* *turgidum* subsp*. dicoccoides* (Körn. Ex Asch. & Graebn.) Thell., *T. turgidum* subsp*. dicoccum* (Schrank) Thell., *T. turgidum* subsp*. durum* (Desf.) Husn. and *T. timopheevii* (Zhuck.) Zhuck. (Danilova et al. 2012). Unlike RFLPs, compensation analysis and chromosome pairing tests, FISH analysis does not require the development of wheat-alien substitution lines or segregating mapping populations; it visualizes homoeologous regions directly on alien chromosomes in a simple and fast experiment.

The purpose of this work was to develop and map a set of FISH cDNA probes specific to wheat homoeologous groups to mark all chromosome arms at distal and proximal positions. The markers were employed in detecting chromosome rearrangements and analyzing homoeologous relationships within wheat genome and genomes of three wild Triticeae species.

## Materials and methods

### Plant material

The material used in this study included *T. aestivum* cv. Chinese Spring TA3008, *Aegilops umbellulata* Zhuk. TA1851, *Ae. caudata* L. TA1908 and the wheat-*Thinopyrum intermedium* (Host) Barkworth and D.R. Dewey translocation line TA5634 homozygous for the translocation chromosome T7BS·7S#3L (Liu et al. 2011) from the collection of the Wheat Genetics Resource Center, Kansas State University. Accession TA1851 was used for developing wheat-*Ae. umbellulata* chromosome addition lines (Friebe et al. 1995; Zhang et al. 1998). Accession TA1908 was used for developing wheat-*Ae. caudata* addition lines (Friebe et al. 1992).

### Slide preparation and FISH procedure

Somatic chromosome preparations using the drop technique, direct probe labeling by nick translation and the FISH procedure were as described previously (Kato et al. 2004, 2006) with minor modifications as described in (Danilova et al. 2012). To make FISH probes, cDNAs were amplified with standard primers T3 and T7; PCR products were purified with Invitrogen PCR purification kit (Life Technologies, Grand Island, NY, USA Cat. # K310001) and labeled with Texas red-5-dCTP (PerkinElmer, Waltham, MA, USA, cat **#** NEL426001EA). GAA- and pAs1- oligonucleotide probes (Danilova et al. 2012) were synthesized by Integrated DNA Technologies with a flourochrome attached to the 5′ end. For nucleolus organizing region (NOR) probe, clone pTa71 was used (Gerlach and Bedbrook 1979). Genomic in situ hybridization (GISH) was performed according to Zhang et al. (2001) with modifications described in Liu et al. (2011). Chromosome preparations were mounted and counterstained with 4′,6-diamidino-2-phenylindole solution (DAPI) or propidium iodide (PI) in Vectashield (Vector Laboratories, Burlingham, CA, USA, cat # H-1200, H-1300). Images were captured with a Zeiss Axioplan 2 microscope using a cooled charge-coupled device camera CoolSNAP HQ2 (Photometrics, Tucson, AZ, USA) and AxioVision 4.8 software (Zeiss). Images were processed using the Adobe Photoshop software (Adobe Systems Incorporated, San Jose, CA, USA).

### FLcDNA map development and chromosome measurements

To develop detectable FISH probes, long (>2.5 kb) wheat FLcDNA sequences (Kawaura et al. 2009) were selected from the Triticeae FLcDNA database (Mochida et al. 2009, http://www.shigen.nig.ac.jp/wheat/komugi/ests/tissueBrowse.jsp). The chromosomal position of cDNAs was detected by BLAST (Altschul et al. 1997) against expressed sequence tags (ESTs) mapped in the deletion bins of Chinese Spring (Qi et al. 2004; GrainGenes Database http://wheat.pw.usda.gov/cgi-bin/westsql/map_locus.cgi) and verified by BLAST against the barley (Deng et al. 2007), rice and *Brachypodium* genome sequences. The cDNAs that showed similarity to ESTs, mapped on more than one homoeologous group were rejected. The presence of repetitive sequences was checked using Repeatmasker software (Smit et al. 1996–2004). The cDNA clones were supplied by the National BioResource Project-Wheat, Japan. *Acc2* cDNA pooled probe was produced from wheat RNA by Reverse Transcriptase-PCR (Danilova et al. 2012). Ten out of 61 FLcDNAs were used in our previous work (Table 1).

**Table 1:** Full length cDNA FISH probes

Chromosome group arm	FISH probe name	FLcDNA, KOMUGI database	cDNA/probe length, bp	Average distance from the centromere			Matching EST	EST position on bin map	FLcDNA position on barley chromosome
				A	B	D			
1S		tplb0028l15	3,037	Signals on 4BS, 4DS, 4AL and background on all chromosomes			BF201704	1AS1-0.47-0.86	4HS (93 %)
								1BS10-0.50-0.84	
								1DS1-0.59-0.70	
	1S-3	AK333586	3,522	0.86 ± 0.03	0.83 ± 0.04 sat^b^	0.78 ± 0.04	BF483372	1AS3-0.86-1.00	1H (93 %, 79 %, 75 %)
								1BS.sat18-0.50-1.00	
								1DS5-0.70-1.00	
	1S-2	AK332649	2,860	0.74 ± 0.05	0.71 ± 0.04 sat	0.79 ± 0.04	BE424845	1AS3-0.86-1.00	1H (93 %)
								1BS.sat18-0.50-1.00	
								1DS5-0.70-1.00	
	1S-1	tplb0048d21^e^	3,487	0.11 ± 0.02	0.07 ± 0.02	0.15 ± 0.01	BE425354	1AS1-0.47-0.86	1H (93 %)
								1DS3-0.48	
1L		tplb0006e16	1,110	Very weak signals on 1L and additional signal on NOR sites			BE407013	1AL1-0.17-0.61	1H (95 %)
								1BL6-0.32-0.47	
								1DL2-0.41-1.00	
	1L-1	tplb0013a02^e^	5,094	0.20 ± 0.03	0.17 ± 0.02	0.18 ± 0.04	BF482555	C-1BL6-0.32	1H (93 %)
	1L-2	tplb0029f23^e^	3,113	0.42 ± 0.03	0.35 ± 0.04	0.38 ± 0.03	BE591501	1BL6-0.32-0.47	1H (94 %)
								1DL2-0.41-1.00	
	1L-3	tplb0014k07	3,872	0.84 ± 0.04	0.79 ± 0.04	0.88 ± 0.03	BE591575	3AL3-0.42-0.78^b^	1H (92 %)
								3BL2-0.22-0.50^b^	
								3DL2-0.27-0.81^b^	
2S	2S-4	tplb0012l12	4,143	0.77 ± 0.02	0.73 ± 0.02	0.71 ± 0.03	BG275030	2AS5-0.78-1.00	2HS (95 %)
								2BS4-0.75-0.84	
								2DS5-0.47-1.00	
	2S-3	AK330599	4,400	0.71 ± 0.04	0.61 ± 0.03	0.63 ± 0.03	BM134309	2AS5-0.78-1.00	2HS (95 %)
								2BS1-0.53-0.75	
								2DS5-0.47-1.00	
	2S-2	AK330434	3,575	0.60 ± 0.03	0.46 ± 0.02	0.48 ± 0.03	BE498640	C-2AS5-0.78	2HS (96 %)
								C-2BS4-0.75	
								2DS5-0.47-1.00	
	4S-4	tplb0043m19	3,384	0.58 ± 0.02	0.46 ± 0.02	0.51 ± 0.07	BG607966	4AS1-0.20-0.63^b^	4HS (96 %) 2HS (90 %)
								C-4BS4-0.37^b^	
								4DS3-0.67-0.82^b^	
	2S-1	tplb0006k18	3,750	0.50 ± 0.03	0.38 ± 0.05	0.37 ± 0.05	BG262632	2DS1-0.33-0.47	2HS (96 %) 7HL (89 %)
2L	2L-1	tplb0007l09	3,165	0.22 ± 0.04	0.13 ± 0.02	0.22 ± 0.05	BG604591	C-2AL1-0.85	2HL (~90 %)
								C-2DL3-0.49	
	2L-2	AK333292	5,191	0.49 ± 0.02	0.32 ± 0.03	0.44 ± 0.02	BE443094	C-2AL1-0.85	2HL (90 %)
	2L-3	tplb0004a16	3,841	0.54 ± 0.02	0.41 ± 0.02	0.45 ± 0.01	BE489940	2BL2-0.36-0.50	2HL (97 %)
								2DL3-0.49-0.76	
	2L-4	AK331687	4,036	0.86 ± 0.04	0.91 ± 0.03	0.89 ± 0.02	BG313362	2AL1-0.85-1.00	2HL (91 %)
								2BL6-0.89-1.00	
								2DL9-0.76-1.00	
3S	3S-4	tplb0001g16	3,127	0.73 ± 0.03	0.66 ± 0.04	0.63 ± 0.02	BE446628	3BS1-0.33-0.57	? (~90 %)
								3DS6-0.55-1.00	
	3S-3	tplb0004j16^e^	4,402	0.58 ± 0.06	0.50 ± 0.05	0.43 ± 0.03	BF202364	3AS4-0.45-1.00	3HS (92 %)
								3DS3-0.24-0.55	
	3S-2	tplb0011e24	1,491	0.42 ± 0.03	0.51 ± 0.04	0.41 ± 0.04	BF200563	3AS4-0.45-1.00	3HS (91 %)
								3BS1-0.33-0.57	
								3DS3-0.24-0.55	
	3S-1	tplb0014n06	3,237	0.41 ± 0.02	0.40 ± 0.05	0.34 ± 0.04	BE446568	C-3AS2-0.23	3HS (96 %)
								3BS1-0.33-0.57	
								3DS6-0.55-1.00	
								6AS1-0.35-0.65	
3L		tplb0007d03	3,316	Produce signals on 3L, and additional brighter signals on 5BS and 4BL			BF201439	3AL3-0.42-0.78	3HL (93 %)
								3BL10-0.50-0.63	
								C-3DL2-0.27	
	3L-1	AK336104	3,860	0.13 ± 0.04	0.06 ± 0.02	0.10 ± 0.02	BE591527	C-3AL3-0.42	3HL (95 %)
								C-3BL10-0.50	
								C-3DL2-0.27	
	3L-2	tplb0045e08	3,369	0.43 ± 0.04	0.33 ± 0.03	0.34 ± 0.02	BE489130	3DL2-0.27-0.81	3HL (86 %)
								3BL2-0.22-0.50	
								3AL3-0.42-0.78	
	3L-3	AK335612	3,596	0.70 ± 0.02	0.66 ± 0.03	0.72 ± 0.04	BG263365	3AL5-0.78-1.00	3HL (92 %)
								3BL7-0.63-1.00	
								3DL2-0.27-0.81	
	*Acc*-*2* ^d, e^		5,592	0.82 ± 0.04	0.82 ± 0.03	0.83 ± 0.04			3HL (96 %) 5HL (90 %)
4BS 4DS 4AL	*Acc*-*2* ^d, e^		5,592	0.69 ± 0.03					
	4S-6	tplb0017g02^e^	3,191	0.53 ± 0.02	0.74 ± 0.03	0.77 ± 0.06	BF485337	C-4AL11-0.66	4HS (95 %)
								4DS2-0.82-1.00	
	4S-5	tplb0001n09	1,602	0.52 ± 0.05	0.73 ± 0.03	0.71 ± 0.07	BF478437	4AL12-0.43-0.59	4HS (~90 %)
								4DS2-0.82-1.00	
	4S-4	tplb0043m19	3,384	0.38 ± 0.03	0.63 ± 0.04	0.62 ± 0.05	BG607966	4AS1-0.20-0.63^b^	4HS (96 %), 2HS (90 %)
								C-4BS4-0.37^b^	
								4DS3-0.67-0.82	
	4S-3	tplb0013i03	4,240	0.33 ± 0.03	0.46 ± 0.05	0.35 ± 0.03	BE606474	4BS4-0.37-0.57^b^	5HL (97 %), 4HS (91 %)
							BE442814	C-4AL12-0.43	
								4BS4-0.37-0.57	
								C-5BL6-0.29	
								C-5DL1-0.60	
	4S-2	tplb0014k23	3,488	0.32 ± 0.04	0.40 ± 0.03	0.35 ± 0.09	BE446722	C-4AL12-0.43	4HS (~97 %), 5HL (89 %)
								C-4DS1-0.53	
4AS 4BS 4DS	4S-1	AK330261	3,582	0.35 ± 0.04	0.37 ± 0.02	0.02 ± 0.07	BE591915	C-4BL1-0.71^b^	4HS (~95 %)
								C-4DL9-0.31^b^	
4BL 4DL 4AS		tplb0006a16	2,184	Contains (CCT)_11_; probe labels repeats^c^					4HL (95 %)
	4L-2	AK335837	3,866	0.21 ± 0.03	0.19 ± 0.03	0.28 ± 0.02	BF484560	4AS1-0.20-0.63	4HL (~90 %)
	4L-3	tplb0033b21^e^	3,024	0.61 ± 0.02	0.44 ± 0.02	0.53 ± 0.02	BE637255	4AS3-0.76-1.00	4HL (94 %)
								4BL1-0.71-0.86^b^	
								4DL9-0.31-0.56	
	4L-4	AK335609	4,790	0.68 ± 0.05	0.47 ± 0.02	0.62 ± 0.05	BE426474	4BL5-0.86-1.00^b^	4HL (96 %)
5S		tplb0018i03	1,623	Contains (CCG)_5_; probe labels repeats^c^			BG314248	5BS5-0.71-0.81	5HL (95 %)
								5DS5-0.67-0.78	
	5S-5	tplb0027f03^e^	2,416	0.79 ± 0.05	0.82 ± 0.03	0.70 ± 0.04	BF482732	3AL3-0.42-0.78^b^	3H (94 %)
								3BL2-0.22-0.50^b^	
								3DL2-0.27-0.81^b^	
	5S-4	tplb0016e11	2,847	0.81 ± 0.01	0.76 ± 0.06	0.69 ± 0.04	BE591734	5AS3-0.75-0.98	5HS (~94 %)
								5BS6-0.81-1.00	
								5DS5-0.67-0.78	
	5S-3	tplb0006h03	3,807	0.77 ± 0.06	0.74 ± 0.04	0.63 ± 0.03	BE495282	5AS3-0.75-0.98	5HS (~93 %)
								5BS5-0.71-0.81	
	5S-2	tplb0002p18	3,112	0.16 ± 0.03	0.24 ± 0.04	0.10 ± 0.02	BE604729	C-5AS1-0.40	5HS (96 %)
								5BS4-0.43-0.56^b^	
								C-5DS1-0.63	
	5S-1	tplb0016k09	3,057	0.08 ± 0.05	0.18 ± 0.02	0.18 ± 0.03	BE606654	C-5AS1-0.40	5HS (93 %)
								C-5BS4-0.43	
								C-5DS1-0.63	
5L		tplb0017c03	4,100	Contains (GGA)_10_; probe labels repeats^c^			BF428826	5AL10-0.57-0.78	5HL (95 %)
								5BL9-0.76-0.79	
								5DL5-0.76-1.00	
	5L-1	tplb0014l23	3,737	0.05 ± 0.01	0.06 ± 0.02	0.07 ± 0.02	BF292055	C-5AL12-0.35	7HS (92 %)
								C-5BL6-0.29	
								C-5DL1-0.60	
	4S-3	tplb0013i03	4,240	0.17 ± 0.02	0.20 ± 0.02	0.16 ± 0.03	BE606474	4BS4-0.37-0.57	5HL (97 %) 4HS (91 %)
							BE442814	C-5BL6-0.29	
								C-5DL1-0.605L	
	5L-2	AK331808	4,808	0.47 ± 0.02	0.46 ± 0.02	0.45 ± 0.03	BE500727	C-5AL10-0.57	5HL (90 %), 2HS (89 %)
								C-5BL14-0.75	
								C-5DL1-0.60	
	5L-3	AK334748	5,408	0.68 ± 0.03	0.62 ± 0.03	0.61 ± 0.03	BF483313	5AL10-0.57-0.78	5HL (94 %)
								5DL5-0.76-1.00^b^	
	5L-4	tplb0043p15^e^	1,514	0.70 ± 0.03	0.74 ± 0.03	0.70 ± 0.02	BF483487 BE404437 BF474029	5DL5-0.76-1.00	5HL (92 %)
								5AL10-0.57-0.78	
	*Acc*-*2* ^d, e^		5,592						
6S		AK331504	3,732	Probe label repeats on 1B, 2B, 4B, 5B			BE498099	C-6DS2-0.45	6HS (95 %)
	6S-2	tplb0006a09	3,685	0.44 ± 0.02	0.31 ± 0.02	0.41 ± 0.02	BF428701	6AS1-0.35-0.65	6HS (90 %)
								6DS2-0.45-0.79	
	6S-1	tplb0050a13	3,244	0.50 ± 0.03	0.34 ± 0.02	0.41 ± 0.03	BM138455	6AS1-0.35-0.65	6HS (85 %)
								6DS2-0.45-0.79	
6L	6L-1	tplb0016o11	2,658	0.89 ± 0.01	0.90 ± 0.02	0.85 ± 0.02	BE403950	6AL8-0.90-1.00	6HL (~95 %)
								6BL	
								5-0.40-1.00	
								6DL10-0.80-1.00	
	6L-2	AK332077	5,017	0.78 ± 0.02	0.76 ± 0.02	0.75 ± 0.03	BG263812	6AL8-0.90-1.00	6HL (95 %)
								6BL5-0.40-1.00	
								6DL11-0.74-0.80	
	6L-3	AK333540	3,938	0.71 ± 0.02	0.67 ± 0.02	0.67 ± 0.03	BM138382	6AL	6HL (95 %)
								6DL12-0.68-0.74	
	6L-4	AK333670	4,377	0.67 ± 0.02	0.62 ± 0.01	0.66 ± 0.03	BF473535	C-6BS5-0.76^b^	6HL (95 %)
								6DS2-0.45-0.79^b^	
	6L-5	tplb0009a09	3,283	0.26 ± 0.02	0.31 ± 0.01	0.25 ± 0.02	BF484691	C-6AL4-0.55	6HL (97 %)
								C-6BL5-0.40	
								6DL6-0.29-0.47	
7S	7S-4	tplb0015e09	3,640	0.71 ± 0.03	0.87 ± 0.02	0.72 ± 0.01	BE445587	7AS8-0.45-0.89	7HS several copies (95 %, 90 %, 85 %, 80 %)
								7DS4-0.61-1.00	
								7BS1-0.27-1.00	
	7S-3	tplb0006n08	3,254	0.63 ± 0.07	0.72 ± 0.04	0.63 ± 0.02	BM138427	7BS1-0.27-1.00	7HS (96 %) 6HL (89 %) 7HL (82 %)
								7DS4-0.61-1.00	
	7S-2	tplb0021a05	2,889	0.63 ± 0.03	0.74 ± 0.04	0.61 ± 0.02	BF293371	C-7BS1-0.27^b^	7HS (95 %)
	7S-1	AK334430	4,404	0.45 ± 0.02	0.51 ± 0.03	0.43 ± 0.03	BE495760		7HS (~ 95 %)
7L	7L-1	tplb0013b07	3,360	0.17 ± 0.04	0.17 ± 0.03	0.13 ± 0.02	BF484535	C-7AL1-0.39	7HL (92 %)
								C-7BL2-0.33	
								C-7DL5-0.30	
	7L-2	tplb0061d08	3,147	0.36 ± 0.03	0.33 ± 0.02	0.27 ± 0.02	BF483178	7AL1-0.39-0.71	7HL (94 %)
	7L-3	tplb0013g03	4,173	0.50 ± 0.02	0.47 ± 0.01	0.46 ± 0.01	BE442845	7BL2-0.33-0.63	7HL (94 %)
								7DL2-0.61-0.82^b^	
	4S-3	tplb0013i03	4,240	0.62 ± 0.03			BE606474	4BS4-0.37-0.57	5HL (97 %) 4HS (91 %)
							BE442814	5L	
	7L-4	tplb0007o14	3,957	0.92 ± 0.02	0.93 ± 0.02	0.90 ± 0.04	BE591418	7AL18-0.90-1.00	7HL (90 %)
								7BL10-0.78-1.00	
								7DL3-0.82-1.00	

^a^Satellite

^b^EST bin position does not correspond well to the FISH signal position

^c^Staining corresponds with repeat distribution reported by (Cuadrado et al. 2008a, b)

^d^ *Acc*-*2* is a pooled probe containing mixture of three RT-PCR products (1,950, 1,942 and 2,078 bp long) (Danilova et al. 2012)

^e^The probe was used in our previous work (Danilova et al. 2012)

Selected cDNAs were used as FISH probes in a mixture with probes to tandem repeats and microsatellites, which allowed chromosome identification. The relative distance of a cDNA FISH site from the centromere was measured and calculated using the MicroMeasure 3.3 software (Reeves and Tear 2000). For measurements, the centromeres were marked on DAPI images of chromosomes as the position of the primary constriction. For most cDNA probes, the signal position was measured on five chromosomes from different metaphase spreads. Average relative distance from the centromere, standard deviation and confidence intervals with a significance level of 0.05 were calculated using Microsoft Office Excel functions. The idiogram was constructed using the wheat standard karyotype (Gill et al. 1991). The heterochromatic N-bands that corresponded to GAA- FISH bands were left on the idiogram and pAs1- FISH bands were added.

## Results

### Mapping FLcDNAs on wheat cv Chinese Spring chromosomes using FISH

**Fig. 1:**
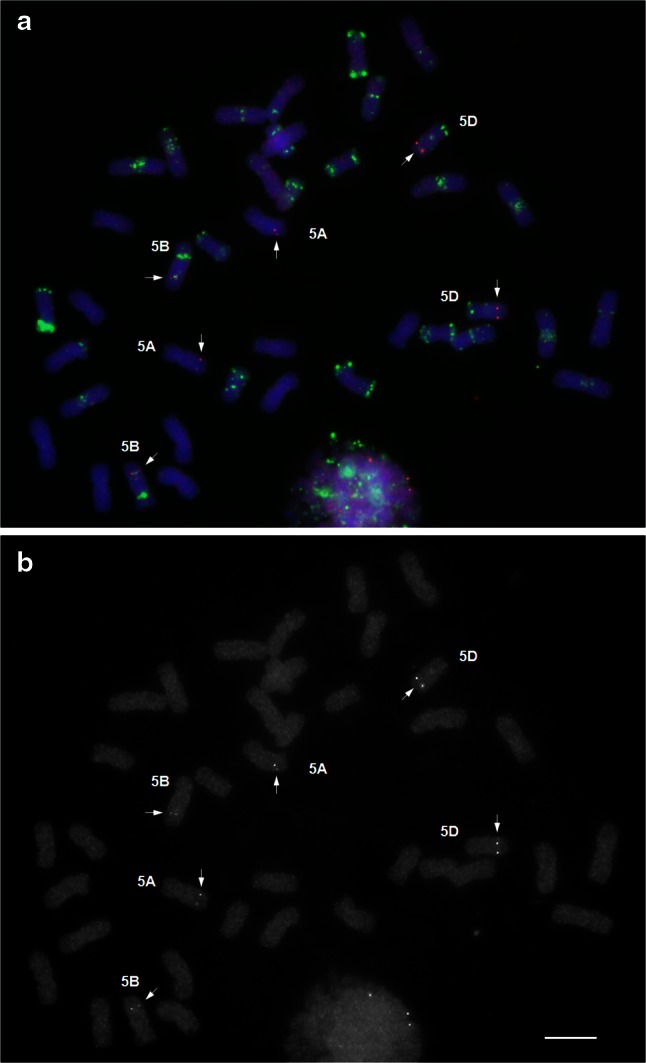
Mapping 5L-3 probe (AK334748) on wheat chromosomes of cv Chinese Spring. **a** Merged image. The cDNA probe is *red*, GAA and pAs1 oligonucleotide probes are *green*, chromosomes, counterstained with DAPI are *blue*. **b** *Red* channel image. The probe hybridizes to three pairs of homoeologous chromosomes. *Bar* corresponds to 10 μm

For each wheat chromosome arm, we selected at least three cDNA clones to develop a distal, interstitial and proximal FISH marker. In total, 61 cDNA sequences were selected to develop FISH probes (Table 1). Fifty-one cDNA probes produced distinguishable FISH signals, no unspecific background and hybridized to all three homoeologous chromosome pairs (Fig. 1). Three cDNA probes hybridized to several homoeologous chromosome sets or on nonhomoeologous chromosomes, 4S-3 (chromosome arms 4BS, 4DS, 4AL and 5AL, 5BL, 5DL and 7AL), 4S-4 (2AS, 2BS, 2DS and 4BS, 4DS, 4AL) and *Acc*-*2* (3AL, 3BL, 3DL, 4AS, 5DL). One probe tplb0006e16 (1,110 bp) was too short and produced a very weak signal on the expected chromosome arm and labeled NOR sites. Six probes showed staining of repeat clusters or unspecific staining on all chromosomes (Table 1). Three cDNAs tplb0006a16, tplb0018i03 and tplb0017c03 contain simple-sequence repeats (CCT)_11_, (CCG)_5_ and (GGA)_10_, respectively, and produced FISH patterns in accordance with the SSRs distribution reported by Cuadrado et al. (2008a). Repeats (GGA)_*n*_/(CCT)_*n*_ are distributed as clusters on specific chromosome sites and repeats (CCG)_*n*_ are dispersed throughout all chromosomes. Three other probes, tplb0028l15, AK331504 and tplb0007d03 label unknown repeats (Supplemental Fig. 1).

**Fig. 2:**
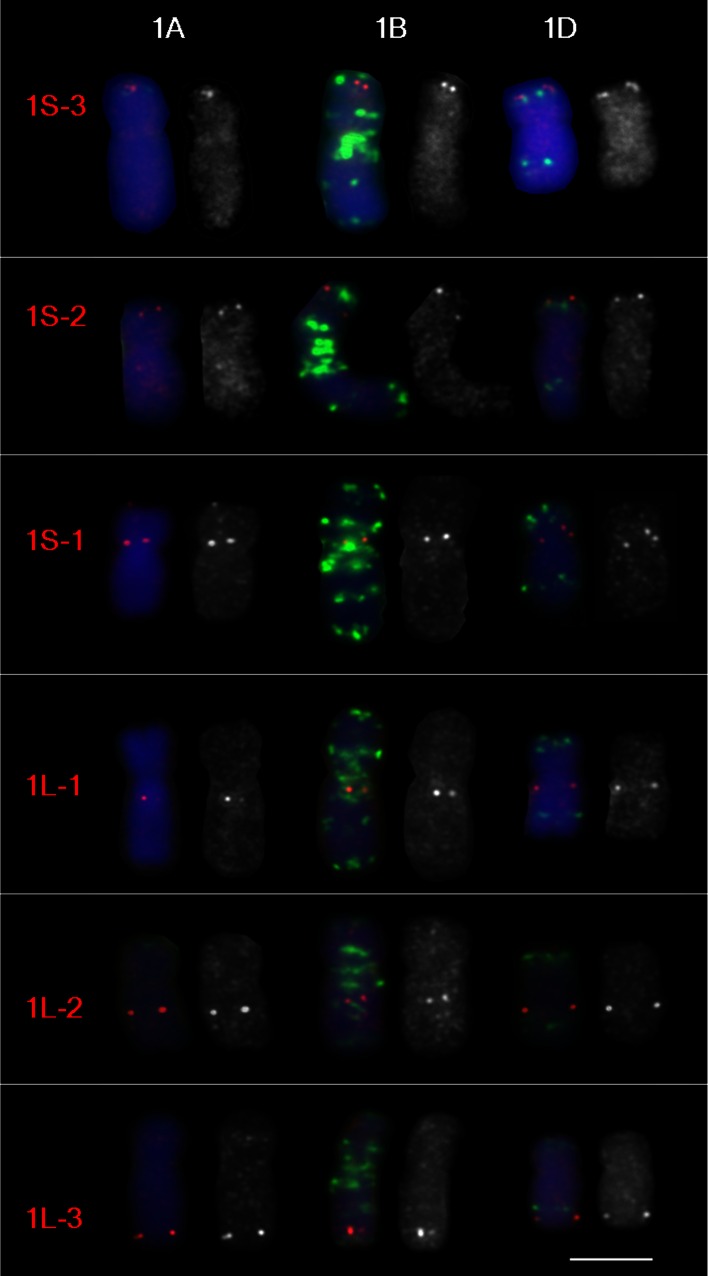
Mapping group-1 cDNA FISH probes on chromosomes of wheat cv Chinese Spring. The cDNA probes are *red*, GAA and pAs1 oligonucleotide probes are *green*, chromosomes counterstained with DAPI are *blue*. Each chromosome is presented as a merged image and a separate *red* channel image. *Bar* corresponds to 5 μm

FISH mapping of group-1 chromosomes is shown in Fig. 2. Some probes showed a difference in the sequence copy number between homoeologs; for example probes 1S-3 and 1S-1 produced double signals on chromosome 1D and probe 1L-3 on chromosome 1A (Fig. 2). Probe 7S-4 hybridizes as a cluster of at least three copies on all group-7 chromosomes and showed a very bright FISH signal (Supplemental Fig. 1).

The FISH positions of 49 cDNAs out of 54 mapped by BLAST against ESTs correlate well with the corresponding EST bin positions (Table 1). The sequence tplb0028l15 matches an EST mapped on the short arms of group-1 chromosomes but is similar to a sequence on chromosome 4HS of barley and was mapped by FISH on chromosome arms 4BS, 4DS and 4AL. The probe 1L-3 (tplb0014k07) was mapped by BLAST against EST sequences on the long arms of group-3 chromosomes, but hybridized to group-1 chromosomes. The cDNA sequence has matches on barley chromosomes 1H (92 % similarity) and 3H (76 %). Probe 6L-4 (AK333670) was mapped by EST similarity on the short arms of group-6 chromosomes, but matches (92 %) to a sequence on barley chromosome arm 6HL and hybridized to the long arms of group-6 chromosomes of wheat. The probe 5S-5 (tplb0027f03) was mapped by EST similarity on the long arms of group-3 chromosomes but hybridized to group 5. This cDNA clone may contain a wrong insert because instead of the expected 2,416 bp, its length is about 3,100 bp. Probe 4S-1 (AK330261) showed FISH signals on the short arms of chromosomes 4A, 4B and 4D. This sequence is 98 % similar to the EST mapped on the proximal part of 4BL and 4DL arms (Table 1), but has 95 % similarity to a sequence mapped on barley chromosome 4HS. For *Acc*-*2* cDNA no matching bin mapped ESTs were found.

**Fig. 3:**
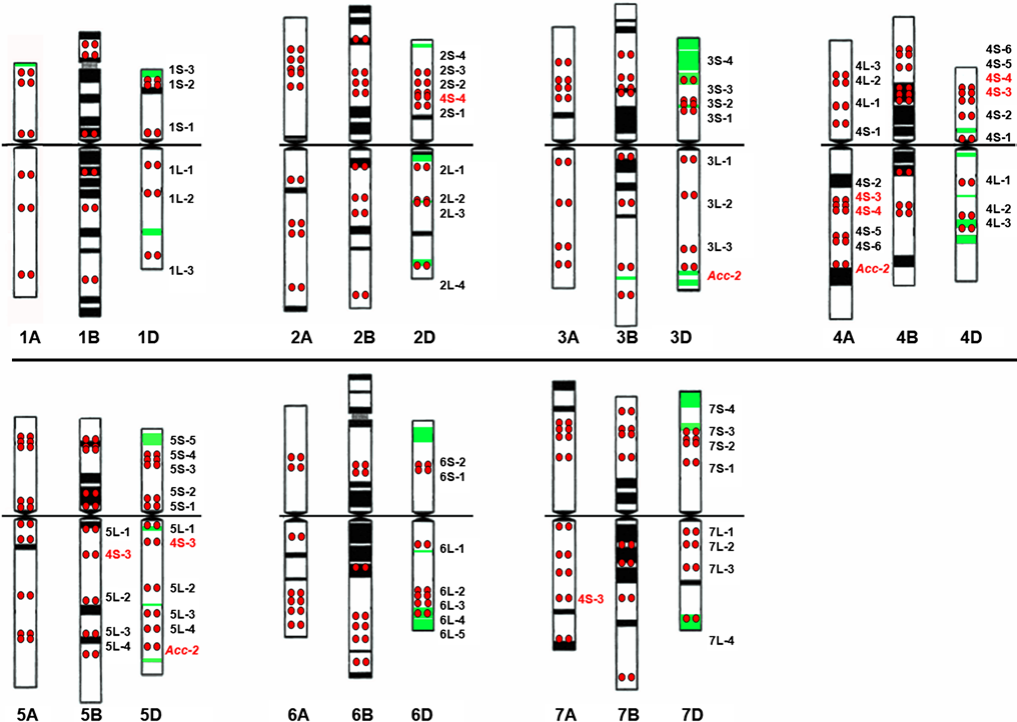
Idiogram of wheat chromosomes. Heterochromatic bands, which can be visualized with GAA oligonucleotide probes, are shown in *black*, pAs1 bands are shown in *green*, cDNA probe positions are shown as *red dots*. The names of the probes, which hybridize to more than one homoeologous group are highlighted in *red*

Most of the cDNA probes hybridized to all three homoeologous chromosomes in the same order and at a similar relative position, except for chromosome arms 2AS, 4A, 4BL, 6AS and 7BS, where the positions of several probes are significantly different from those of the other wheat homoeologs (Table 1; Fig. 3). On chromosome 4A, known to be rearranged (Naranjo et al. 1987; Devos et al. 1995; Mickelson-Young et al. 1995; Miftahudin et al. 2004), all three group-4 long arm specific cDNA probes hybridized to the short arm of 4A and five cDNAs mapped on short arm of group-4 chromosomes hybridized to the long arm of 4A. One exception is probe 4S-1 (AK330261), which showed FISH signals on the short arms of all group-4 chromosomes.

The positions of the cDNA FISH probes, GAA and pAs1 bands are shown on the idiogram (Fig. 3). There are three or more FISH markers on the distal, interstitial and proximal positions on the long arms of all chromosomes except those of group 4. The cDNA FISH markers are distributed unequally along the short arms of most chromosomes and those of group 4, leaving some parts uncovered. The short arm of group -6 chromosomes has markers only at interstitial positions. Thus, we did not find markers at desirable positions for some chromosome arms, because there were either no long cDNAs with similarity to any physically mapped EST found in the Triticeae FLcDNA database or the ESTs were mapped to a very large bin, but corresponding cDNAs hybridized at adjacent positions, as on short arms of chromosome groups 5, 6 and 7.

### FISH on chromosomes of Triticeae species

**Fig. 4:**
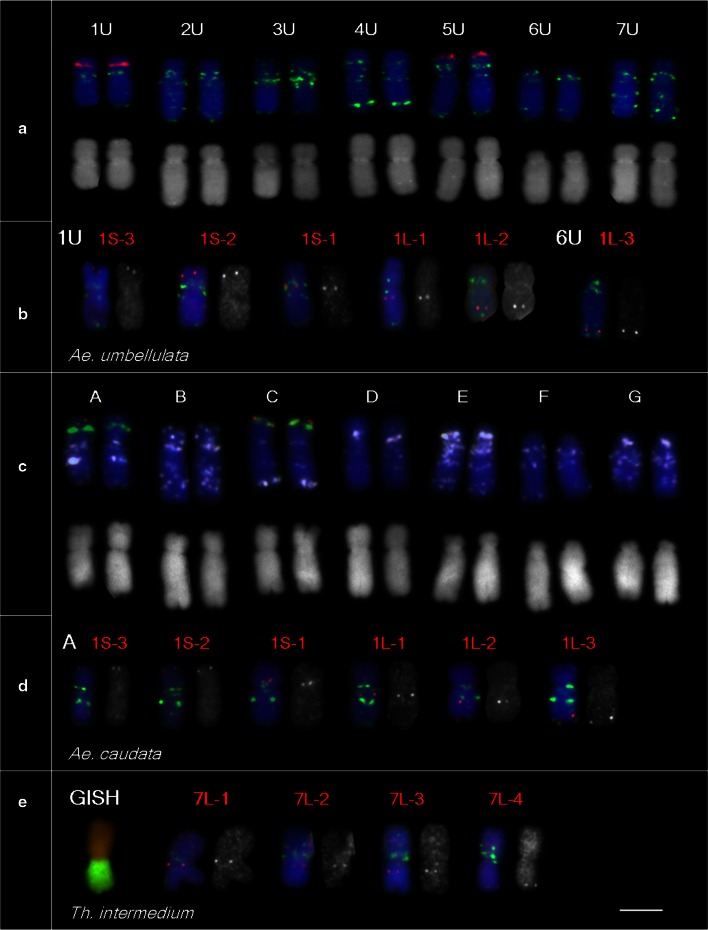
Application of wheat cDNA FISH probes to three Triticeae species: *Ae. umbellulata* (**a**, **b**), *Ae. caudata* (**c**, **d**) and *Th. intermedium* (**e**). **a**, **c** Chromosomes of *Ae. umbellulata* and *Ae. caudata* were identified by FISH pattern of tandem repeats and chromosome morphology; **a** GAA is *green*, NOR is *red*, **c** GAA is *white*, NOR is *green*, pAs1 is *red*. Karyotypes were constructed with chromosomes from a single metaphase. **b**, **d** Group-1 chromosome-specific probes are *red*, the GAA repeat is *green*. **e** The translocation chromosome T7BS 7S#3L with 7BL substituted by 7S#3L of *Th. intermedium* was identified by GISH (*Th. intermedium* gDNA is *green*, wheat DNA, counterstained with PI, is *red*) and by GAA pattern (*green*). Group-7 chromosome-specific probes are *red*. *Bar* corresponds to 5 μm

To test whether wheat probes produce detectable signals on chromosomes of other Triticeae species and reveal chromosome rearrangements, we selected *Ae. umbellulata* as a model. Its genetic map was constructed using RFLP markers and homoeologous relationships with wheat were studied by Zhang et al. (1998). The genetic map provided evidence for multiple translocations in *Ae. umbellulata* chromosomes relative to those of hexaploid wheat. At first, we developed a FISH karyotype of *Ae. umbellulata* by labeling GAA microsatellites and NOR sites. All chromosomes were identified (Fig. 4a) by comparing their FISH pattern, size and arm ratio with the C-banding karyotype (Friebe et al. 1995). Then, each of six wheat probes, specific for group-1 chromosomes, was applied to *Ae. umbellulata* preparations together with the GAA oligonucleotide probe. All wheat cDNA probes produced distinct FISH signals on *Ae. umbellulata* chromosomes; five were detected on chromosome 1U at similar positions and order as on wheat group-1 chromosomes (Fig. 4b). Probe 1L-3, located on the distal end of the long arms of wheat group-1 chromosomes, was detected on the distal end of chromosome arm 6UL. According to Zhang et al. (1998), chromosome 1U is colinear with most of wheat chromosome 1D except the distal segment, which was translocated to the distal end of 6U. Thus, our results correspond with those of the RFLP analysis.

We applied the same approach and the same set of probes to *Ae. caudata*, where the chromosome homoeology to wheat is not known. All chromosomes of *Ae. caudata* were identified (Fig. 4c) by comparing their GAA-FISH pattern, positions of NOR sites and morphology with C-banding karyotype developed by Friebe et al. (1992). The six wheat cDNA probes produced clear signals on chromosome A of *Ae. caudata* in the same order and similar positions as those on wheat chromosomes of group 1 (Fig. 4d), conferring its homoeology and suggesting that this chromosome is not rearranged.

*Thinopyrum intermedium* was another Triticeae species with an unknown chromosome structure selected for the FISH analysis. We have developed a line with the translocation chromosome T7BS·7S#3L, which confers resistance to wheat streak mosaic and *Triticum* mosaic virus (Liu et al. 2011). We employed induced homoeologous recombination (reviewed in Qi et al. 2007) to shorten the 7S#3L segment, however, after screening 400 progenies, no recombinants were recovered. This result prompted us to verify the colinearity of chromosome arm 7S#3L. The translocation chromosome was identified by its GAA pattern in which the 7BS arm has specific bands and the 7S#3L arm has no GAA bands. The four wheat 7L-specific cDNA probes produced clear signals on the *Th. intermedium* chromosome arm in the same order and positions as those on the long arms of wheat group-7 chromosomes (Fig. 4d), indicating no rearrangements.

## Discussion

### FISH map of wheat chromosomes

The coding sequences of wheat genomes are highly conserved; mean synteny estimates between the A-, B- and D-genome loci, based on Southern blot analysis of ESTs collection, ranged from 0.91 to 0.96 (Akhunov et al. 2003). Although it is not known from which genome of *T. aestivum* originate the FLcDNAs used in our experiment, 51 out of 61 cDNAs hybridized to all three homoeologous chromosomes and can be used as homoeologous group specific wheat markers. Seven probes contain repetitive sequences and label dispersed or tandem repeats and three other probes hybridize to more than one homoeologous or nonhomeologous chromosome (Table 1); consequently, some chromosome arms are missing FISH markers at desirable positions.

For 91 % cDNAs hybridized to all three homoeoloci, the positions of FISH site correspond to the matching EST positions on bin deletion map. The disagreement between EST and FISH maps may be caused by presence of paralogous genes. The small size probes used in Southern analysis to map ESTs (Qi et al. 2004) may detect sequences shared by several genes. An example is sequence tplb0014k07 (probe 1L-3) which matches to sequences on barley chromosomes 1H (92 % similarity) and 3H (76 %) and to wheat EST mapped on group-3 chromosomes, but produced FISH signal on group-1 chromosomes (Table 1). Some wheat deletion chromosomes may be rearranged or telosomics may be actually acrocentrics which may cause errors in mapping of pericentromeric regions (probe 4S-1). For all cDNAs mapped, their position on wheat chromosomes corresponds to positions of homoeoloci on barley sequence physical map (The International Barley Genome Sequencing Consortium 2012). Thus, our FISH map can be integrated with the wheat deletion bin map and together with the barley sequence physical map can help to verify mismapped wheat loci.

The homoeologous genomes of *T. aestivum* were found to be largely collinear, i.e. composed of homoeoloci at equivalent positions along the chromosomes, except chromosomes 2B, 4A, 5A, 6B and 7B involved in translocations (Devos et al. 1993b, 1995; Mickelson-Young et al. 1995; Dvorak 2009). The order and location of the cDNA FISH probes on the homoeologous genomes were colinenar as well (Table 1; Fig. 3), except chromosome arms 2AS, 4A, 4BL, 6BS, and 7BS. The proximal displacement of two probes mapped on the 6BS arm may be explained by overestimation of the arm length because of the large size of the secondary constriction at the NOR site (Dvorak et al. 1984). Chromosome 4A is known to be rearranged as a result of 4AL/5AL translocation followed by a 7BS translocation and one paracentric and two pericentric inversions (Naranjo et al. 1987; Devos et al. 1995; Mickelson-Young et al. 1995; Miftahudin et al. 2004), and all but one group-4 cDNA probe hybridized accordingly. The difference in the position of probe 4S-1 on the short arm of chromosomes 4B and 4D and the proximal displacement of three cDNA probes on 4BL (Table 1; Fig. 3) can be explained by a pericentric inversion, specific for chromosome 4B of Chinese Spring and some other cultivars (Endo and Gill 1984; Friebe and Gill 1994). Probe 4S-1 hybridized to the short arm of chromosome 4A which may result from an additional pericentric inversion. The displacement of all four 7BS FISH probes to the distal end of 7BS reflects the translocation of a large part of chromosome arm 7BS to 4AL. Devos et al. (1993b) found that the distal end of chromosome arm 2BS was deleted as a result of 2BS/6BS translocation. We found that on chromosome arm 2AS the positions of all five cDNA probes differ significantly from 2BS and 2DS, they all displaced distally. We found the same displacement on Chinese Spring deletion bin map: among 100 EST probes physically mapped to 2AS5-0.78-1.00 bin, 20 are mapped to more proximal bins of 2BS1-0.53-0.75, and 4 mapped to 2DS1-0.33-0.47 bin (http://wheat.pw.usda.gov/cgi-bin/westsql/map_locus.cgi). For mapping chromosome arm 2DS a stock with very large distal deletion was used (1–0.47), which makes it less informative. Based on our measurements and EST mapping, we assume that 2AS has either a distal deletion or a translocation in cv Chinese Spring. Thus, our data confirm previously known wheat chromosomal rearrangements and can be used to detect the new rearrangements.

### Application of wheat FISH markers in studying chromosome homoeology of Triticeae

The success of gene transfer by induced homoeologous recombination (Riley et al. 1968; Sears 1977) depends on genome affinity and chromosome colinearity. A significant difference in meiotic pairing of alien chromosomes or chromosome arms with wheat homoeologs correspond to the level of structural rearrangements (Ceoloni et al. 1988; Cuadrado et al. 1997; Devos et al. 1993a; Lukaszewski et al. 2004). The lack of knowledge on the evolutionary and cytogenetic relationship among wheat and its relatives hampers the alien gene transfer or causes non-compensating translocations, which is the reason why only few alien genes were intensively being used in cultivar improvement (Friebe et al. 1996; Ceoloni and Jauhar 2006). RFLP analysis was so far the most informative approach to study homoeology of wheat and alien chromosomes and to reveal rearrangements. Cytogenetic studies of Triticeae employ a large collection of tandem repeats, which allow to identify individual chromosomes. But because the abundance and distribution of the repeats is highly variable among species, they are not suitable for detecting chromosome homoeology, unless applied in chromosome pairing analysis. We developed wheat cytogenetic map using FISH with probes specific to coding regions of homoeologous chromosomes. Earlier we showed that wheat FLcDNA probes can be used to detect homoeologous chromosomes and reveal rearrangements in genomes of closest wheat diploid and tetraploid relatives *T. urartu* and *T. monococcum* (genome AA), *Ae. speltoides* (SS), *Ae. tauschii* (DD), *T.* *turgidum* subsp*. dicoccoides*, *T. turgidum* subsp*. dicoccum*, *T. turgidum* subsp*. durum* (AABB) and *T. timopheevii* (AAGG) (Danilova et al. 2012). In the current study we extended this approach to more distant wheat relatives *Ae. umbellulata,* *Ae. caudata* and *Th. intermedium*.

*Aegilops umbellulata* and *Ae. caudata* are annual, diploid, self-pollinated grasses. *Aegilops umbellulata* (2*n* = 14, UU) belongs to *Aegilops* L. section which includes nine species of different ploidy level; all of them have the U-genome combined with one of C, M, N or S genomes. *Aegilops caudata* (2*n* = 14, CC) belongs to section Cylindropyron (Jaub. et Spach) Zhuk., which has only one other species, *Ae. cylindrica* Host (2*n* = 28, DC) (Kilian et al. 2011). *Thinopyrum intermedium* is a perennial, cross-pollinating, allohexaploid species (2*n* = 6*x* = 42, genome JJJ^s^J^s^SS); the J genome is related to *Th. bessarabicum* (Savul. and Rayss) A. Löve, J^s^ is related to *Dasypyrum villosum* L. Candargy and S is related to a *Pseudoroegneria*-like progenitors (Chen et al. 1998; Liu et al. 2011; Mahelka et al. 2011). Phylogenetic analyses of several genic regions showed that sequences of *Aegilops* and *Triticum* species, including the C and U genomes, are similar and form a tight cluster, separate from *Pseudoroegneria* species and *D. villosum*. Within the *Aegilops*-*Triticum* clade of diploid species, the U and C genomes form a tighter subcluster, which shows their high similarity (Kellogg et al. 1996; Mason-Gamer 2005; Petersen et al. 2006; Escobar et al. 2011; Mahelka et al. 2011). Based on the phylogenetic data, we can assume that the genic sequences of *Ae. umbellulata*, *Ae. caudata* and other *Aegilops* and *Triticum* species are similar enough to be detected by wheat FISH probes. Indeed, the hybridization of wheat chromosome 1 -specific cDNA probes to their chromosomes was successful. FISH confirmed the rearrangement of *Ae. umbellulata* chromosome 1U, detected earlier by RFLP analysis (Zhang et al. 1998). The homoeologous relationships of *Ae. caudata* chromosomes to wheat have not been studied previously, although this species is an important source of many agronomic traits (Friebe et al. 1992; Makkouk et al. 1994; Riar et al. 2012). Our data revealed that chromosome A of *Ae. caudata* is homoeologous to wheat group-1 chromosomes and is not involved in any major rearrangements.

In spite of the large phylogenetic distance and dissimilarity between wheat and S-genome of *Th. intermedium*, wheat cDNA probes were detected on the 7S#3L chromosome arm. FISH mapping did not reveal any structural rearrangements, hence, our failure in recovering any recombinant chromosomes can be explained by low affinities of evolutionary distant genomes, which was confirmed by chromosome pairing analysis. The frequency of meiotic pairing between chromosome arms 7S#3L and 7BL in plants, homozygous for mutant gene *ph1b* (Sears 1977), was very low −0.3 %, (B. Friebe, T.V. Danilova, unpublished data), indicating, that for recovering recombinants with shortened *Th*. *intermedium* segments, a larger population needs to be screened.

The successful detection of wheat cDNA probes on all three wheat homoeologs and on chromosomes of eight Triticeae species and the high sequence similarity between wheat and barley homoeoloci (90 % or more) allows us to expect, that FISH with probes specific to genic regions can be used to detect chromosome homoeology and rearrangements in Triticeae. We plan to map additional FISH markers on wheat chromosomes to increase the resolution of this approach.

## Electronic supplementary material

Below is the link to the electronic supplementary material.

Supplemental Fig. 1. a, b) Probe tplb0007d03 label unknown repeats on chromosomes 5B and 4B. c, d) Probe 7S-4 (tplb0015e09) labels a gene cluster on short arms of group-7 chromosomes. BLAST analysis showed 90 % similarity of tplb0015e09 to *Brachypodium distachyon* and *Setaria italica* ABC transporter C family member 10-like mRNA and to *Zea mays* multidrug-resistance associated protein 3 (MRP3) gene. a, c) Merged images; cDNA probe is red, GAA and pAs1 oligonucleotide probes are green, chromosomes, conterstained with DAPI are blue. b, d) Red channel image. Bar corresponds to 10 μm. (TIFF 60245 kb)
