# Supplementary figures and images for: Development of a wheat single gene FISH map for analyzing homoeologous relationship and chromosomal rearrangements within the Triticeae
Source: Theor Appl Genet. 2014 Jan 10;127(3):715–30. doi: 10.1007/s00122-013-2253-z (PMC3931928; doi:10.1007/s00122-013-2253-z)

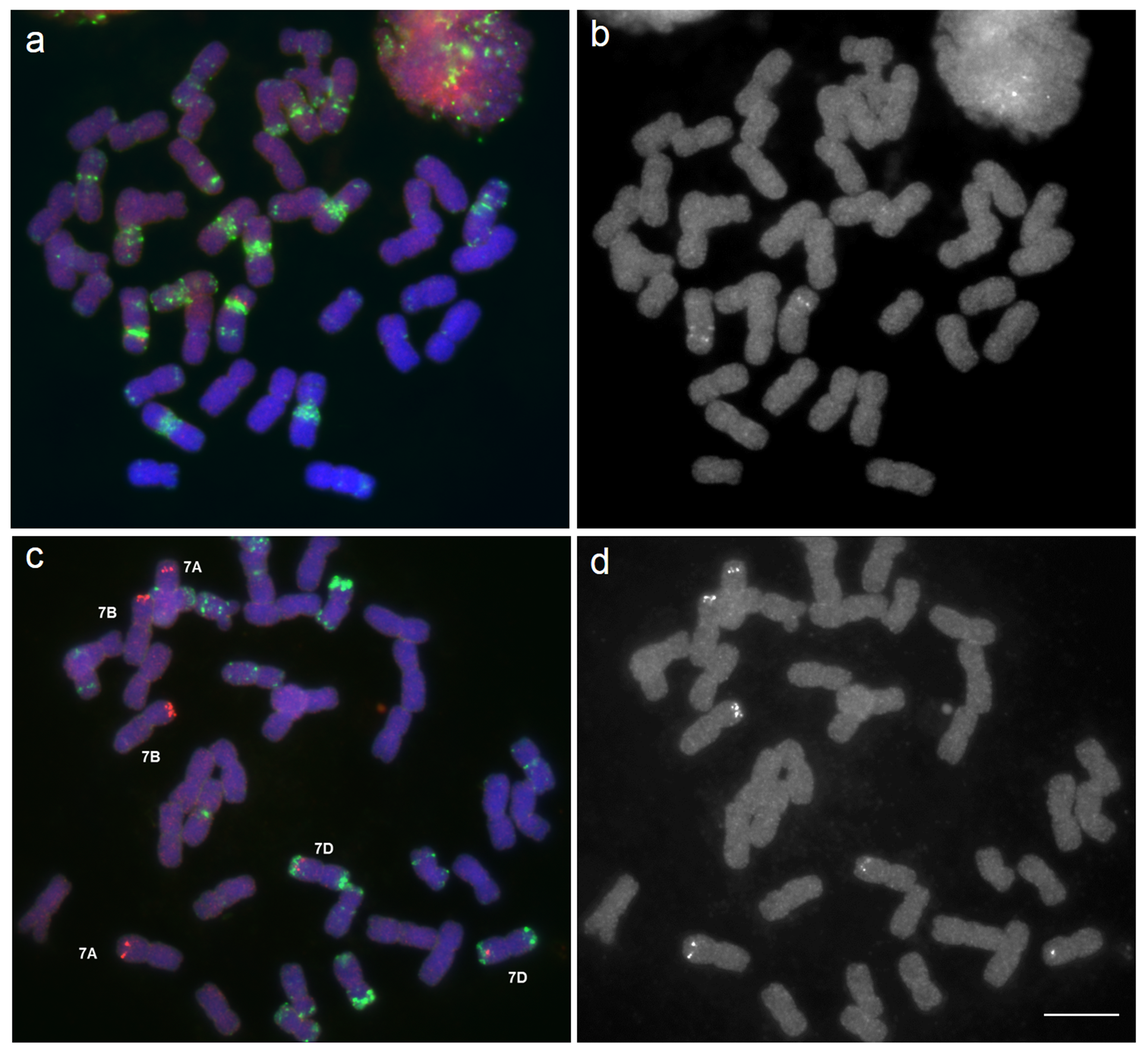

Supplement: Supplementary file 1 — Supplemental Fig. 1. a, b) Probe tplb0007d03 label unknown repeats on chromosomes 5B and 4B. c, d) Probe 7S-4 (tplb0015e09) labels a gene cluster on short arms of group-7 chromosomes. BLAST analysis showed 90 % similarity of tplb0015e09 to Brachypodium distachyon and Setaria italica ABC transporter C family member 10-like mRNA and to Zea mays multidrug-resistance associated protein 3 (MRP3) gene. a, c) Merged images; cDNA probe is red, GAA and pAs1 oligonucleotide probes are green, chromosomes, conterstained with DAPI are blue. b, d) Red channel image. Bar corresponds to 10 μm. (TIFF 60245 kb) [file 122_2013_2253_MOESM1_ESM.tif]
